# Surveillance Bias in Cancer Risk After Unrelated Medical Conditions: Example Urolithiasis

**DOI:** 10.1038/s41598-017-08839-5

**Published:** 2017-08-14

**Authors:** Kari Hemminki, Otto Hemminki, Asta Försti, Kristina Sundquist, Jan Sundquist, Xinjun Li

**Affiliations:** 1 Division of Molecular Genetic Epidemiology, German Cancer Research Center (DKFZ), Im Neuenheimer Feld 580, D-69120 Heidelberg, Germany; 2 Center for Primary Health Care Research, Lund University, 205 02 Malmö, Sweden; 3 Department of Urology, Helsinki University Hospital, Helsinki, Finland; 4 Cancer Gene Therapy Group, Faculty of Medicine, University of Helsinki, Helsinki, Finland; 5 Stanford Prevention Research Center, Stanford University School of Medicine, Stanford California, 94305-5705 USA

## Abstract

We analysed cancer risks in patients with urinary tract stones but some features of the generated results alarmed us about possible surveillance bias, which we describe in this report. We used nationwide Swedish hospital records to identify patients with urinary tract stones (N = 211,718) and cancer registration data for cancer patients for years 1987 to 2012. Standardized incidence ratios (SIRs) for cancer were calculated after the last medical contact for urinary tract stones. All cancers were increased after kidney (SIR 1.54, 95%CI: 1.50–1.58), ureter (1.44, 1.42–1.47), mixed (1.51, 1.44–1.58) and bladder stones (1.63, 1.57–1.70). The risk of kidney cancer was increased most of all cancers after kidney, ureter and mixed stones while bladder cancer was increased most after bladder stones. All SIRs decreased steeply in the course of follow-up time. Tumour sizes were smaller in kidney cancer and *in situ* colon cancers were more common in patients diagnosed after urinary tract stones compared to all patients. The results suggest that surveillance bias influenced the result which somewhat surprisingly appeared to extend past 10 years of follow-up and include cancers at distant anatomical sites. Surveillance bias may be difficult to avoid in the present type of observational studies in clinical settings.

## Introduction

Surveillance bias is a type of information bias which occurs when one group of subjects is followed up more closely than others, for example, if they undergo medical treatment^1, 2^. The term is often used synonymously with detection bias, particularly, if the bias is due to the use of a particular diagnostic technique or type of equipment. A typical setting for surveillance bias is diagnostic work-out for a symptomatic disease leading to a fortuitous finding of an unrelated disease that may be asymptomatic. For example, Craig and Feinstein reviewed 43 studies on second primary cancers and pointed out that only 5 considered the possibility of surveillance bias^1^. In cancer screening programs detection bias leads to some degree of over-diagnosis which is tolerated because of overall net clinical benefits^3^.

The likelihood of surveillance bias has increased because diagnostic tests are more commonly used and in many areas of medicine imaging techniques, such as computed tomography, ultrasound and magnetic resonance imaging have allowed visualisation of structures that were not seen before, for example early cancers^4, 5^. Urolithiasis (UL, urinary tract stone disease) patients are examined by computed tomography for diagnosis of kidney, ureter and mixed stones. Patients with bladder stones commonly receive cystoscopy and palpation of the prostate, and all UL patients may additionally be imagined by thorax radiography. Even if there is alertness about surveillance bias it may be limited by medical speciality and by risk factors for disease considered in the context of that speciality, and the full medical history of patients in terms of unrelated comorbidities may often remain unknown. Cancer registries provide reliable diagnostic data in countries, such as Sweden, where practically all diagnosed cases are reported with histological or cytological verification^6^. However, many cancers develop slowly, some within a decade or more, from a precursor lesion through an *in situ* stage into cancer with metastatic potential^7^. Thus diagnostic and screening procedures may find cancers at an asymptomatic stage which may be of lower grade than symptomatic cancer but still a histologically verified malignancy.

The early aim of the present study was to assess the risk for urological cancers in patients who had previously been diagnosed with UL. UL is a common disease affecting up to 15% of population and many patients have a recurrent disease^8, 9^. UL is thought to be associated with the risk of kidney and bladder cancers, and a study from Taiwan’s National Health Insurance Research Database reported that UL is associated with a high risk of many systemic cancers in addition to urinary tract cancers^10–12^. Thus we considered analysing all cancer types after UL diagnosis. In the present study we use nation-wide Swedish hospital records of inpatients and outpatients with UL diagnoses and link the individual data to cancer data. We analyse the data in terms of follow-up time, proportion of *in situ* tumours and tumour size. In the course of the analyses the likelihood of detection bias became apparent, and the aims were shifted towards featuring some typical signs of such a bias.

## Results

The total number of patients diagnosed with UL during years 1987 to 2012 was 211,718, distributed by the most common type, ureter stones (91,397, 43.2%), followed by kidney stones (77,972, 36.8%), mixed stones (23,890, 11.3%) and bladder stones (18,459, 8.7%). We analysed the risk of 8 specific cancers, all other cancers together and all cancers combined after the last identified medical contact for the four types of UL (Table 1). All cancers were rather uniformly increased after kidney (SIR 1.54), ureter (1.44), mixed (1.51) and bladder stones (1.63). The risk of kidney cancer was increased most after kidney (3.75), ureter (2.78) and mixed stones (4.01) while bladder cancer was increased most after bladder stones (3.34). After bladder stones, SIRs were highest for prostate (1.74) and colon (1.60) cancers. Remarkably, all the listed cancers were increased in patients with kidney and ureter stones. Cancers at anatomically distant sites, in the lung and the breast, were increased after all types of UL, except for lung cancer after bladder stones.

**Table 1:** SIR for cancer of patient with urolithiasis, 1987–2012.

Cancer site	Kidney				Ureter				Mixed				Bladder			
	O	SIR	95% CI		O	SIR	95% CI		O	SIR	95% CI		O	SIR	95% CI	
Colon	486	**1.54**	**1.40**	**1.68**	725	**1.46**	**1.35**	**1.57**	108	**1.38**	**1.13**	**1.67**	208	**1.60**	**1.39**	**1.83**
Pancreas	154	**1.96**	**1.66**	**2.30**	209	**1.70**	**1.48**	**1.95**	39	**2.06**	**1.46**	**2.81**	38	1.33	0.94	1.83
Lung	438	**1.51**	**1.37**	**1.66**	581	**1.24**	**1.15**	**1.35**	96	**1.36**	**1.10**	**1.66**	129	1.14	0.95	1.36
Breast	482	**1.26**	**1.15**	**1.38**	604	**1.38**	**1.28**	**1.50**	141	**1.44**	**1.21**	**1.70**	44	**1.53**	**1.11**	**2.05**
Prostate	1311	**1.34**	**1.27**	**1.42**	2416	**1.37**	**1.32**	**1.43**	331	**1.30**	**1.16**	**1.45**	874	**1.74**	**1.62**	**1.86**
Kidney	311	**3.75**	**3.34**	**4.19**	375	**2.78**	**2.50**	**3.07**	82	**4.01**	**3.19**	**4.98**	64	**2.15**	**1.66**	**2.75**
Urinary bladder	422	**2.15**	**1.95**	**2.37**	589	**1.73**	**1.60**	**1.88**	113	**2.35**	**1.93**	**2.82**	350	**3.34**	**3.00**	**3.71**
Melanoma	300	**1.41**	**1.25**	**1.58**	473	**1.45**	**1.33**	**1.59**	68	1.16	0.90	1.47	73	1.26	0.99	1.59
Other sites	2998	**1.54**	**1.48**	**1.59**	4223	**1.42**	**1.38**	**1.46**	762	**1.51**	**1.40**	**1.62**	986	**1.41**	**1.33**	**1.51**
All	6902	**1.54**	**1.50**	**1.58**	10195	**1.44**	**1.42**	**1.47**	1740	**1.51**	**1.44**	**1.58**	2766	**1.63**	**1.57**	**1.70**

Bold type: 95% CI does not include 1.00.

O = observed number of cases; SIR = standardized incidence ratio; CI = confidence interval.

In Table 2 cancer risk after any UL diagnosis was assessed by follow-up time since the last UL diagnosis. For all cancer the SIR was 4.70 during the first year (4239 cases) but then stabilised to 1.31 (12,159 cases) and 1.24 (5208 cases) during 1–9 and 10 + years of follow-up, respectively. The initial SIRs were excessive, 22.40 and 14.02 for kidney and bladder cancers but they were high also for prostate (4.89) and pancreas (4.80) cancers. All but lung cancers were increased even at the latest follow-up time: risk for kidney cancer remained the highest (1.56) while non-urological cancers showed SIRs between 1.22 and 1.38.

**Table 2:** SIR for cancer of patient with urolithiasis, 1987–2012.

	Follow-up (years)											
	<1				1–9				10+			
Cancer site	O	SIR	95% CI		O	SIR	95% CI		O	SIR	95% CI	
Colon	243	**3.91**	**3.44**	**4.44**	888	**1.36**	**1.28**	**1.46**	396	**1.28**	**1.16**	**1.42**
Pancreas	79	**4.80**	**3.80**	**5.99**	267	**1.62**	**1.43**	**1.83**	94	**1.38**	**1.12**	**1.69**
Lung	171	**2.87**	**2.45**	**3.33**	773	**1.26**	**1.18**	**1.36**	300	1.12	0.99	1.25
Breast	146	**2.46**	**2.08**	**2.89**	769	**1.27**	**1.18**	**1.36**	356	**1.28**	**1.15**	**1.42**
Prostate	1029	**4.89**	**4.60**	**5.20**	2602	**1.17**	**1.13**	**1.22**	1301	**1.22**	**1.16**	**1.29**
Kidney	400	**22.40**	**20.26**	**24.70**	366	**1.68**	**1.52**	**1.87**	112	**1.56**	**1.29**	**1.88**
Urinary bladder	596	**14.02**	**12.92**	**15.20**	614	**1.39**	**1.28**	**1.50**	264	**1.30**	**1.15**	**1.46**
Melanoma	99	**2.39**	**1.94**	**2.91**	556	**1.32**	**1.21**	**1.43**	259	**1.35**	**1.19**	**1.52**
Other sites	1473	**3.76**	**3.57**	**3.95**	5324	**1.34**	**1.31**	**1.38**	2126	**1.22**	**1.17**	**1.28**
All	4236	**4.70**	**4.56**	**4.84**	12159	**1.31**	**1.29**	**1.33**	5208	**1.24**	**1.21**	**1.28**

Bold type: 95% CI does not include 1.00.

O = observed number of cases; SIR = standardized incidence ratio; CI = confidence interval.

As surveillance may lead to a shift in earlier stages we compared the proportion of *in situ* cancers in UL and all cancer patients among patients diagnosed during 2002 to 2012 because the Tumor, Node, Metastasis (TNM) classification was introduced in year 2002 in the Cancer Registry. We compared patients diagnosed at the age 50 + years because most UL related cancers were diagnosed in this age group. *In situ* cancers were reported essentially only for colon and breast cancers and for melanoma. The only significant difference was for colon cancer for which 254/1249 (20.3%) UL related cases were *in situ* forms compared to 7624/42,666 (17.9%, p = 0.025) for all colon cancers. Tumour size migration was assessed by comparing the proportion T0 and T1 to all defined T classes, diagnosed during the first year after UL diagnosis. The only significant difference was for kidney cancer for which 106/176 (60.2%) were stages T0 + T1 of all defined T cases in UL related cancers compared to 3353/7303 (45.9%, p =  < 0.001) in all kidney cancer.

Patients diagnosed with UL in the Hospital Discharge Register during 1987 to 1999 were followed for UL related visits in the Outpatient Register during 2001 to 2012. Most patients (4680, 79.2%) had no UL related visits in the Outpatient Register and their overall cancer risk was 1.13 (1.09–1.16); 20.8% of the patients had at least one visit, and their cancer risk was not increased (0.98, 0.93–1.14).

## Discussion

Based on the results in Table 1 a noncritical interpretation would point out that UL is associated with cancer at multiple sites, even though the affected organs appeared to convey the highest risk. Considering the high prevalence of UL in the population, a logical conclusion should have been that UL is associated with a high population burden for cancer. The follow-up results in Table 2 exposed the concerns about surveillance bias, which may to be due to incidental clinical findings by computed tomography in diagnosis of kidney, ureter and mixed stones. Bladder stones are diagnosed by cystoscopy and palpation of the prostate, which may explain the high risks of prostate and colon cancers. All UL patients may be imagined by thorax radiography which may reveal tumours in the covered parts of the body.

It was somewhat surprising that the bias appeared to extend past 10 years of follow-up. The reason may be that in many patients UL is recurrent or at least may increase concerns leading to frequent medical contacts^8, 9^. However, using the Outpatient data to follow UL patients for multiple UL related visits, only 20.9% had one or more visits, suggesting that many UL patients with multiple episodes were seen in the primary care for which we have no data. Also the data showed that the simple number of Outpatient visits for UL causes did not appear to explain cancer risks because even patients with no additional visits showed an excess risk of 1.13.

The more frequent *in situ* diagnoses and smaller tumour sizes In UL patients compared to all cancer patients supported the existent of surveillance bias although the differences were modest. The mechanism of UL related cancer, in humans as well as in experimental animals, is believed to be mechanical wear and inflammation^10–12^. Yet it would not be self-evident that UL would cause systemic cancers because usually inflammation, for example, by autoimmune diseases is associated with cancers at sites affected by inflammation^13–16^.

Although the above data suggest involvement of surveillance bias, we need to admit that we have not eliminated alternative mechanisms which predispose to UL and which might jointly predispose to cancer. Stone formation is due to a combination of genetic and environmental factors. Risk factors include high urine calcium levels, obesity, certain foods, some medications, calcium supplements, hyperparathyroidism, gout, diabetes, hypertension and not drinking enough fluids^9^. Among these obesity and diabetes are known risk factors of cancer, and kidney cancer, showing the highest risk in the present study, is one of the highest obesity-related cancers^17–19^.

Whether it is possible to eliminate surveillance bias by a study design may not be simple. A frequently applied method is the omission of early follow-up but this may lead to removal of true cases if these are properly diagnosed, such as histologically verified cancers. Comparison of cancer risk between early and late onset UL and in persons suffering from multiple episodes should be informative but not bias-free. We conclude that quantification of risks may be extremely difficult in situations when surveillance bias is likely, and scientists and readers of scientific texts should be aware of such problems in observational studies.

## Methods

UL patients were identified using the nationwide Swedish Hospital Discharge Register (1986–2012) and the Outpatient Register (2001–2012). The first UL diagnosis in either register was included and a patient was only entered once. Information from the registers was linked at the individual level via the national 10-digit civic registration number to the Swedish national Cancer Registry. In the linked dataset, civic registration numbers were replaced with serial numbers to ensure the anonymity of all individuals. Revisions 9 (1987–1996) and 10 (1997-) of the International Classification of Diseases (ICD) was used to identify UL diagnostic codes. Only 54,500 patients were diagnosed during the ICD-9 period, compared to 166,600 in the ICD-10 period. Standardized incidence ratios (SIRs) were calculated as the ratio of observed to expected number of cases. The follow-up was started from the last UL diagnosis and ended at diagnosis of the first primary cancer, death (Causes of Death Register) or end of follow-up, December 31, 2012, whichever came first. The expected numbers were calculated for all individuals without a history of UL (i.e., essentially for the whole Swedish population covered by the Database), and the rates were standardized by 5-year-age, gender, period (5 years group), socioeconomic status and residential area. The 95% confidence interval (95%CI) of the SIR was calculated assuming a Poisson distribution. Data on the TNM classification were available from year 2002 onwards.

In order to assess the frequencies of medical contacts due to UL and their influence on SIRs we identified patients who were diagnosed with UL using the Hospital Discharge Register during 1987 to 1999 and followed their UL related visits from the Outpatient Register during 2001–2012.

### Ethical statement

The study was approved by the Ethical Committee of Lund University and the study was conducted in accordance with the approved guidelines not requesting informed consent. The study is national register-based study on anonymous personal data.
